# Case Report: A clinically relevant isolation of *Gardnerella leopoldii* guided by morphological and molecular evidence from a urinary tract infection case

**DOI:** 10.3389/fmed.2025.1548067

**Published:** 2025-04-02

**Authors:** Liangyou Chen, Wei Weng, Dan Li, Weipeng Xie, Lingling Lu, Shuo Li

**Affiliations:** ^1^Department of Urology, Affiliated Jinhua Hospital, Zhejiang University School of Medicine, Jinhua, China; ^2^Precision Medicine Center, Affiliated Jinhua Hospital, Zhejiang University School of Medicine, Jinhua, China; ^3^Department of Clinical Laboratory, The Second Hospital of Jinhua, Jinhua, China; ^4^Zhejiang Key Laboratory of Digital Technology in Medical Diagnostics, Dian Diagnostics Group Co., Ltd., Hangzhou, China

**Keywords:** urinary tract infection (UTI), *Gardnerella leopoldii*, metagenomic next-generation sequencing (mNGS), microscopic morphology, case report

## Abstract

**Background:**

The genus *Gardnerella* is commonly found in the vaginal ecosystem and is considered a covert pathogen of the urinary tract. However, *Gardnerella vaginalis* had been the only recognized species of the genus *Gardnerella* for decades. Cases regarding the clinical relevance of *Gardnerella leopoldii* have rarely been reported, which is crucial for fully understanding the various species within the genus *Gardnerella*.

**Case presentation:**

A 72-year-old female patient was admitted to the hospital with gross hematuria and complaints of waist soreness. Physical examinations, including those of the head, chest, and abdomen, along with routine laboratory tests such as white blood cell (WBC) count and proportion, liver function, and renal function, yielded normal results. However, the patient also exhibited significantly elevated levels of serum C-reactive protein (CRP) and abnormal urinary test findings, which revealed positive results for occult blood and leukocyte esterase, and increased counts of erythrocyte and leukocyte. To further evaluate the urinary system, computerized tomography urography (CTU) was performed. The CTU results revealed multiple weakly enhanced foci in the right kidney and thickening of the right ureter, renal pelvis, calyces, and bladder walls. Based on the above findings, the initial diagnosis included hematuria, hydronephrosis, and urinary tract infection (UTI). To identify the causative pathogens, we employed a comprehensive approach that included microscopic morphology, Sanger sequencing, and metagenomic next-generation sequencing (mNGS). Finally, both *Mycobacterium tuberculosis* and *G. leopoldii* were identified as the co-infecting etiological agents responsible for the patient’s urinary tract infection.

**Conclusion:**

This case represents the first documented isolation of clinically relevant *G. leopoldii*, guided by morphological and molecular evidence from a clinical urine sample. It highlights the potential of mNGS as a promising tool for identifying previously unrecognized species and offers valuable insights to enhance the understanding of clinically relevant microorganisms.

## 1 Introduction

The genus *Gardnerella* has been extensively reported as a key component of polymicrobial biofilms (1) and is strongly associated with bacterial vaginosis in women (2, 3). Although the genus *Gardnerella* is commonly found in both the vagina and urine (4), emerging research suggests a relationship between the presence of *Gardnerella* in urine and recurrent urinary tract infections (UTIs). Accordingly, the clinical significance of *Gardnerella* spp. may be underestimated, and its role as a potential covert pathogen in urine warrants further investigation (5). For nearly four decades, *Gardnerella vaginalis* was the only recognized species within the genus *Gardnerella* (6). However, in 2019, *Gardnerella leopoldii*, *Gardnerella swidsinskii*, and *Gardnerella piotii* were delineated separately for the first time (6, 7) due to technical advancements in whole-genome sequencing analysis. Therefore, previous studies on UTIs and *Gardnerella* have mainly focused on *G. vaginalis*. There have been limited reports on the clinical relevance of *G. leopoldii*, which is essential for a comprehensive understanding of the relationship between different species of *Gardnerella* and their potential associations with clinical conditions.

Herein, we present a case of a female patient with UTI and gross hematuria. Through the use of gold-standard culture, microscopic morphology, and molecular detection, the causes of UTI were identified as a co-infection of *Mycobacterium tuberculosis* and *G. leopoldii*. To the best of our knowledge, this is the first clinically relevant isolation of *G. leopoldii*, which could be essential for enhancing the understanding of the species characteristics of the *Gardnerella* genus.

## 2 Case presentation

A 72-year-old female patient was admitted to the hospital for 1 day due to gross hematuria and a primary complaint of waist soreness. Initial evaluation at the outpatient clinic using B-mode ultrasound revealed signs of right hydronephrosis. Upon physical examination, no apparent abnormalities were detected in the chest or abdomen, but positive percussion pain was observed in the right renal area.

**Figure 1:**
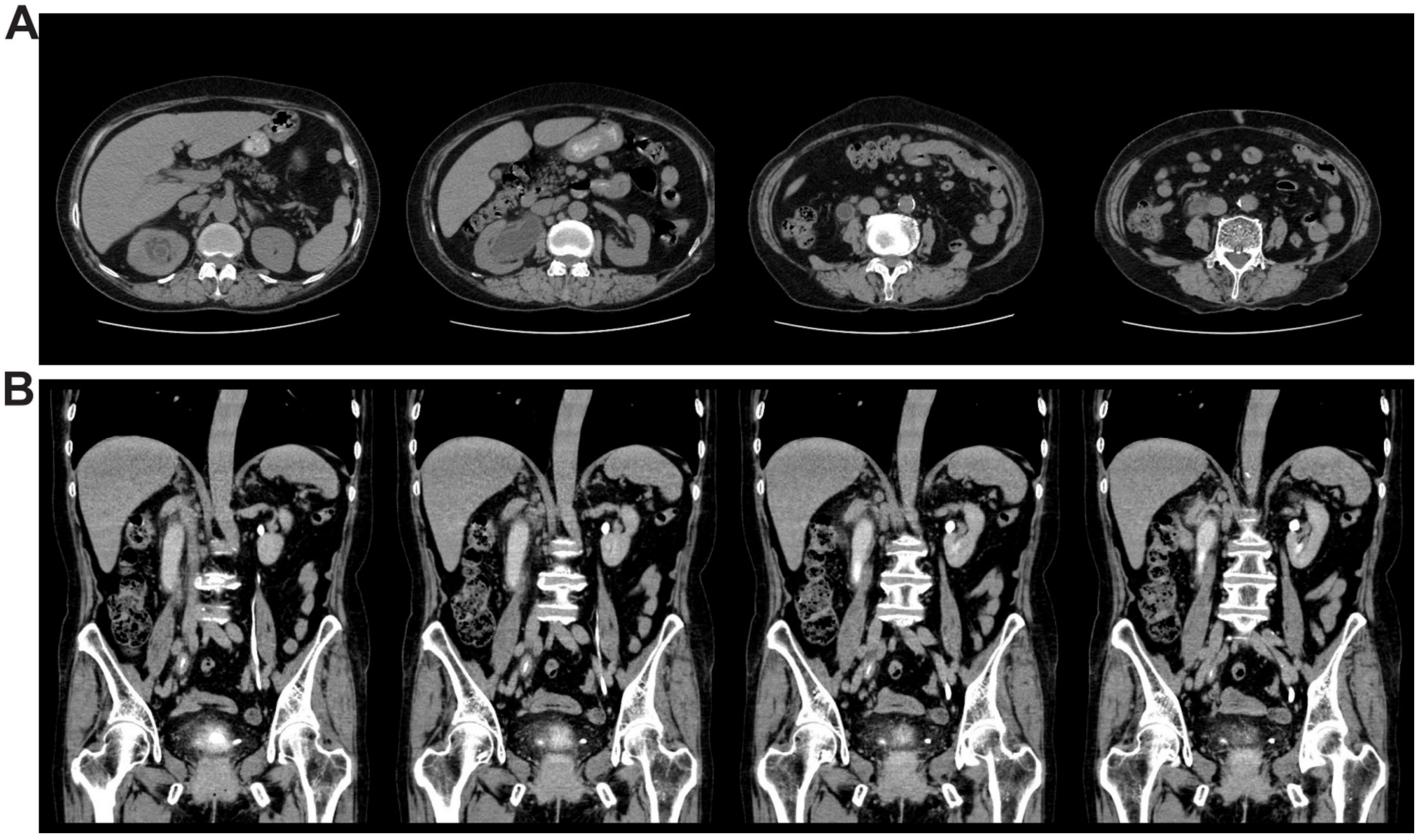
CT images showed thickening and hydronephrosis of the right kidney and right ureter, as well as inflammation of the bladder. **(A)** Whole abdominal CT of the patient. **(B)** CT urography of the patient.

Laboratory tests were also performed, revealing the following results. Routine blood tests indicated a white blood cell (WBC) count of 5.92 × 10^9^/L (within the normal range of 3.5–9.5 × 10^9^/L), with a proportion of 64.7% categorized as neutrophils (normal range 40–75%). Serum C-reactive protein (CRP) levels were found to be elevated at 35.50 mg/L (normal range 0–6.00 mg/L). Liver function indicators were found to be normal. Urinalysis revealed 3+ for occult blood and leukocyte esterase, with an elevated urinary erythrocyte count of 84.5/μL (normal range 0–25.0/μL), a markedly high urinary leukocyte count of 20103.1/μL (normal range 0–30.0/μL), and an increased epithelial cell count of 63.1/μL (normal range 0–21.4/μL). The glomerular filtration rates (GFR, normal range >90.0 mL/min) were measured at 25.32 mL/min for the right kidney, indicating moderate function with reduced excretion, and 36.38 mL/min for the left kidney, suggesting normal function with slightly delayed excretion. Given the suggestive finding of localized inflammation on the outpatient ultrasound, a computerized tomography (CT) scan was performed. Chest CT revealed mixed ground-glass nodules in the upper lobe of the right lung, multiple small nodules in the left lung, and evidence of emphysema in both lungs. Abdominal CT revealed a gross bladder wall, thickening of the right ureter, renal pelvis, and calyces, right-sided hydronephrosis and ureter dilation, multiple enlarged retroperitoneal lymph nodes, and gallbladder stones (Figure 1A). CT urography (CTU) further confirmed hydronephrosis of the right kidney and ureter, the presence of a cyst in the right kidney, and inflammation of the bladder (Figure 1B). Based on previous clinical experience, the initial diagnosis was hematuria and hydronephrosis secondary to a urinary tract infection (UTI).

**Figure 2:**
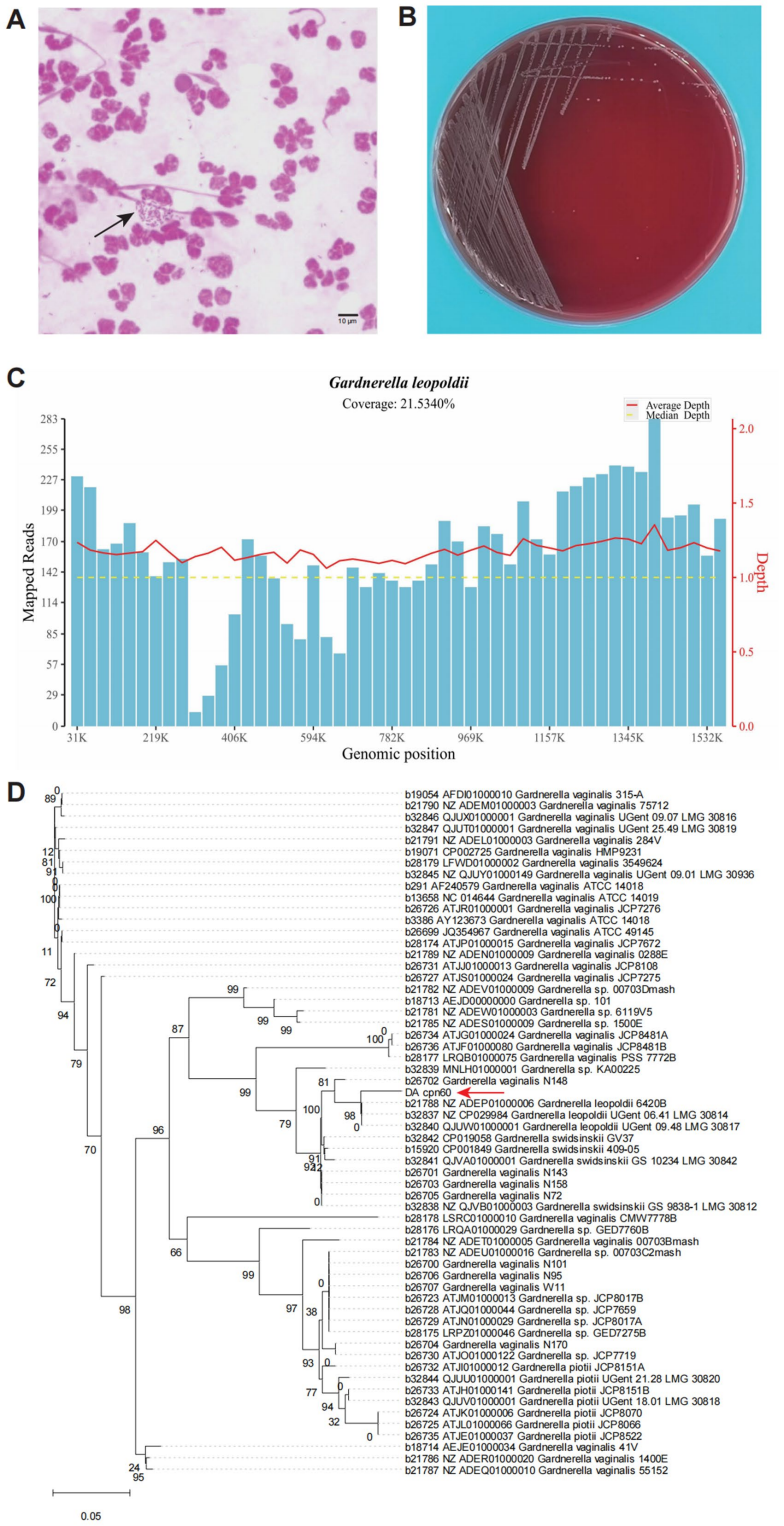
Morphological and molecular evidence revealed a urinary tract infection caused by *G. leopoldii*. **(A)** Microscopic field of the urinary smear exhibited Gram-negative coccobacilli internalized by neutrophils (black array marked). **(B)** Colonies growing on a blood agar plate, which were later molecularly confirmed as *G. leopoldii*. **(C)** Genome coverage distribution of the mapped reads sequenced using mNGS. **(D)** Phylogenetic tree derived from the *cpn60* gene sequences of the isolate in this study (DA_cpn60, red array marked) and others of *Gardnerella* spp. from cpnDB (https://www.cpndb.ca/).

Considering the complexity of microbial colonization in the urinary tract, a clean-catch midstream urine sample was collected for pathogen identification. DNA pellets from the urine sample were extracted for metagenomic next-generation sequencing (mNGS). Meanwhile, microbiological detection, including culture and smear examinations, was also conducted. The microscopic examination of the stained urine smear revealed coccobacilli being phagocytosed by immune cells (Figure 2A), confirming a bacterial infection in the urinary tract (8). White colonies were successfully cultured on a blood agar plate (Figure 2B) and were initially reported as *G. vaginalis* using the VITEK^®^ Mass Spectrometry Identification System (Bio Mérieux, France).

To confirm the identification, colony DNA was extracted using the TIANamp Bacteria DNA Kit (TIANGEN Biotech, China) for Sanger sequencing of the 16S rRNA and *chaperonin60* (*cpn60*) genes. BLAST analysis of the 16S rRNA gene amplicon sequence revealed a high similarity to both *G. leopoldii* and *G. vaginalis* (Table 1), according to the National Center for Biotechnology Information (NCBI, https://blast.ncbi.nlm.nih.gov/Blast.cgi). For mNGS sequencing, we utilized the Universal Plus DNA Library Prep Kit for MGI (GeneDian, China) to prepare the libraries, which were then sequenced with MGISEQ200 (BGI, China). However, the mNGS results revealed *Mycobacterium tuberculosis* complex (2 reads) and *G. leopoldii* (1,832 reads) (Table 2). The genomic coverage of the mapped reads (Figure 2C) and the phylogenetic tree of the *cpn60* gene (Figure 2D) confirmed the isolates as *G. leopoldii*, rather than *G. vaginalis*.

**Table 1:** BLAST results of the 16S rRNA gene sequences of the isolated single colony (top 10 BLAST in the NCBI).

No.	Description	Scientific name	Max score	Query cover	Percent identity	Accession length	Accession number
1	*Gardnerella leopoldii* strain UGent06.41 16S ribosomal RNA, partial sequence	*Gardnerella leopoldii*	2,580	100%	99.79	1,524	NR_171541.1
2	*Gardnerella leopoldii* strain UGent 06.41 chromosome	*Gardnerella leopoldii*	2,580	100%	99.79	1,563,545	CP029984.1
3	Uncultured bacterium clone rRNA201 16S ribosomal RNA gene, partial sequence	uncultured bacterium	2,575	100%	99.72	1,508	AY958974.1
4	*Gardnerella* sp. strain c31Ua_26 16S ribosomal RNA gene, partial sequence	*Gardnerella* sp.	2,573	100%	99.72	1,474	OP402842.1
5	Uncultured bacterium clone rRNA416 16S ribosomal RNA gene, partial sequence	Uncultured bacterium	2,564	100%	99.57	1,506	AY959189.1
6	Uncultured bacterium clone rRNA258 16S ribosomal RNA gene, partial sequence	Uncultured bacterium	2,564	100%	99.57	1,479	AY959031.1
7	Uncultured bacterium clone rRNA119 16S ribosomal RNA gene, partial sequence	Uncultured bacterium	2,564	100%	99.57	1,502	AY958892.1
8	*Gardnerella vaginalis* strain JPC8481B 16S ribosomal RNA gene, partial sequence	*Gardnerella vaginalis*	2,560	99%	99.71	1,410	JX860321.1
9	Uncultured bacterium clone rRNA218 16S ribosomal RNA gene, partial sequence	Uncultured bacterium	2,558	100%	99.5	1,479	AY958991.1
10	*Gardnerella vaginalis* strain GS10234 16S ribosomal RNA gene, partial sequence	*Gardnerella vaginalis*	2,556	100%	99.5	1,523	MH898659.1

**Table 2:** Pathogenic microorganisms identified using mNGS in the urine specimen.

Genus	Species	Category	Number of detected reads
*Mycobacterium*	*Mycobacterium tuberculosis* complex	Bacteria	2
*Gardnerella*	*Gardnerella leopoldii*	Bacteria	1,832
*Prevotella*	*Prevotella timonensis*	Bacteria	34
*Enterococcus*	*Enterococcus faecalis*	Bacteria	34
*Ureaplasma*	*Ureaplasma urealyticum*	Mycoplasma	24
*Betapolyomavirus*	Human polyomavirus 1	Virus	8
*Lymphocryptovirus*	Human gammaherpesvirus 4	Virus	2

In addition, the results of the GeneXpert MTB/RIF assay and interferon-gamma release assay (IGRA) confirmed the diagnosis of tuberculosis. Although urogenital tuberculosis (UGTB) could explain hematuria, the presence of phagocytosed bacteria suggested *G. leopoldii* was also clinically relevant. Therefore, the UTI was attributed to a co-infection of *M. tuberculosis* and *G. leopoldii*. The sequencing data from Sanger sequencing and mNGS were submitted to the China National GeneBank Database (CNGBdb) (9) under accession numbers CNP0004870 and CNP0005073.

Metronidazole (400 mg/8 h p.o.) was empirically administered for 7 days to treat urinary anaerobic bacterial infections. After confirming the co-infection of *M. tuberculosis* and *G. leopoldii*, the patient was transferred to a specialized tuberculosis hospital due to regulatory requirements for tuberculosis treatment. After a 4-month follow-up, the patient is currently receiving active triple anti-tuberculosis therapy with isoniazid, rifampicin, and ethambutol.

## 3 Discussion

Herein, we present a case of urinary tract co-infection with *M. tuberculosis* and *G. leopoldii*. Notably, this could be the first documented clinically relevant isolation of a pathogenic strain of *G. leopoldii*, identified through microscopic morphology and molecular detection.

The genus *Gardnerella* was first reported in 1980 as a significant contributor to puerperal infections (10). Subsequent studies have found *Gardnerella* as a core component of the urinary microbiota in postmenopausal women (11) and ranked it as the second most commonly detected genus in both vaginal and urine samples (4). Interestingly, although *Gardnerella* was not a dominant urinary microbe in premenopausal women (11), women in a younger cohort exhibited a higher prevalence and abundance of *Gardnerella* in urine samples from the urethra compared to the older cohort (12). *Gardnerella* could be detected in the urine samples of women with low absolute but high relative abundance. Based on high-throughput sequencing of the 16S rRNA gene, pathogenic *Gardnerella* has been reported to be associated with bacterial vaginosis in women (13) and with severe complications following UTIs in both men (13) and women (14), especially involving *G. vaginalis*. The presence of *Gardnerella* in the urinary tract is considered a risk factor for UTIs and is increasingly recognized as a covert pathogen (5). In this case, the mNGS-detected reads of *Gardnerella* were over 10-fold higher compared to the reads of *Prevotella* and *Enterococcus* (Table 1). Furthermore, the microscopic examination of the urinary staining smear demonstrated immune phagocytosis of the microbes (Figure 2A), providing evidence of a local infection (8). However, the staining results of the microbes revealed them to be Gram-negative, which seemed inconsistent with the taxonomic status of *G. leopoldii*. This discrepancy can be attributed to the special cell wall structure of *G. leopoldii*, which can result in variable Gram staining, despite its taxonomic classification as a Gram-positive bacterium (7). Based on the above evidence, we believe that the genus *Gardnerella* was proliferating abnormally and acting as an opportunistic pathogen rather than as a commensal microorganism in this patient.

For nearly four decades, *G. vaginalis* was the only recognized species in the genus *Gardnerella*, possibly due to the high genetic similarity among species, as was initially suspected in this case. However, advancements in molecular techniques have led to the identification and classification of additional species, including *G. leopoldii*, *G. swidsinskii*, and *G. piotii*, which were delineated as distinct species in 2019 (7, 15). Studies on *G. leopoldii* have mainly focused on genetic heterogeneity and taxonomic diversity within the *Gardnerella* genus (6, 7) or the resolution and co-occurring patterns in the vaginal microbiome (16). Cases of clinically relevant *G. leopoldii* remain scarce. In this study, the Gram staining of the urine smear revealed Gram-negative coccobacilli internalized by neutrophils. Using molecular detection techniques, including Sanger sequencing and mNGS, the coccobacilli were definitively identified as *G. leopoldii*. This case suggests that the presence of urinary *G. leopoldii* could be pathogenic under specific circumstances, such as co-infection with urinary tuberculosis.

Urogenital tuberculosis (UGTB) is typically caused by *M. tuberculosis* or *Mycobacterium bovis*, which leads to infectious inflammation of the urogenital system. Clinical classifications of UGTB mainly include tuberculosis of the kidney, urinary tract, and genital region (17). In addition, disseminated tuberculosis could also affect the urogenital system (17, 18). Symptoms of UGTB include frequent voiding, dysuria, pyuria, back or abdominal pain, and microscopic or macroscopic hematuria (19). In cases of disseminated tuberculosis, systematic symptoms may also rise, such as malaise, fever, and anorexia (20). A history of *M. tuberculosis* infection (17), particularly pulmonary tuberculosis (21), is a significant risk factor for UGTB. Other risk factors include acquired immunodeficiency syndrome (AIDS) (19) and recurrent UTIs (17). UGTB can affect all ages, although it is rare in patients under the age of 20 years because of the long latent period (20). Due to the non-specific presentation and limited sensitivity of a single test to diagnose tuberculosis, a precise diagnosis requires the performance of multiple tests. Therefore, previous publications on urogenital tuberculosis primarily address the challenges of challenges, treatment options, and clinical features (22–24). To the best of our knowledge, only one article reported a co-infection of *Mycoplasma genitalium* and *Chlamydia trachomatis* in an infertile female patient with genital tuberculosis (25). In this case study, urinary tract infection caused by both *M. tuberculosis* and *G. leopoldii* was reported. The clinical team found no evidence of acquired immunodeficiency or a previous history of UTIs; however, the CT images did show a mixed ground-glass nodule in the right lung and a nodular area of increased density in the left lung, both with clear margins. The patient also reported a long history of pulmonary tuberculosis spanning several decades, which may contribute to renal symptoms and coinfections with *G. leopoldii*.

## 4 Conclusion

In conclusion, this case illustrates the first documented isolation of clinically relevant *Gardnerella leopoldii*, which was identified as a coinfection with *M. tuberculosis*. In addition, it highlights the utility of mNGS for the rapid and accurate identification of complicated UTIs. It is believed that mNGS technology is a promising tool for identifying and guiding the discovery of previously unrecognized species. When combined with conventional microbiological methods, mNGS can enhance the discovery and understanding of clinically relevant species, as exemplified by the identification and characterization of *G. leopoldii* in this case.

## Data Availability

The datasets presented in this study can be found in online repositories. The names of the repository/repositories and accession number(s) can be found below: https://db.cngb.org/cnsa/, CNP0004870, https://db.cngb.org/cnsa/, CNP0005073.
